# Bio-Guided Fractionation of Stem Bark Extracts from *Phyllanthus muellarianus*: Identification of Phytocomponents with Anti-Cholinesterase Activity

**DOI:** 10.3390/molecules26144376

**Published:** 2021-07-20

**Authors:** Marina Naldi, Gloria Brusotti, Gabriella Massolini, Vincenza Andrisano, Caterina Temporini, Manuela Bartolini

**Affiliations:** 1 Department of Pharmacy and Biotechnology, Alma Mater Studiorum University of Bologna, Via Belmeloro 6, 40126 Bologna, Italy; marina.naldi@unibo.it; 2 Centre for Applied Biomedical Research—CRBA, University of Bologna, St. Orsola Hospital, Via Massarenti 9, 40138 Bologna, Italy; 3 Department of Drug Sciences, University of Pavia, Viale Taramelli 12, 27100 Pavia, Italy; gloria.brusotti@unipv.it (G.B.); gabriella.massolini@unipv.it (G.M.); caterina.temporini@unipv.it (C.T.); 4 Department for Life Quality Studies, Alma Mater Studiorum University of Bologna, Corso D’Augusto 237, 47921 Rimini, Italy; vincenza.andrisano@unibo.it

**Keywords:** *Phyllanthus muellarianus*, bioassay-guided fractionation, anticholinesterase activity, anti-amyloid properties

## Abstract

A combination of flash chromatography, solid phase extraction, high-performance liquid chromatography, and in vitro bioassays was used to isolate phytocomponents endowed with anticholinesterase activity in extract from *Phyllanthus muellarianus.* Phytocomponents responsible for the anti-cholinesterase activity of subfractions PMF1 and PMF4 were identified and re-assayed to confirm their activity. Magnoflorine was identified as an active phytocomponent from PMF1 while nitidine was isolated from PMF4. Magnoflorine was shown to be a selective inhibitor of human butyrylcholinesterase—hBChE (IC_50_ = 131 ± 9 μM and IC_50_ = 1120 ± 83 μM, for hBuChE and human acetylcholinesterase—hAChE, respectively), while nitidine showed comparable inhibitory potencies against both enzymes (IC_50_ = 6.68 ± 0.13 μM and IC_50_ = 5.31 ± 0.50 μM, for hBChE and hAChE, respectively). When compared with the commercial anti-Alzheimer drug galanthamine, nitidine was as potent as galanthamine against hAChE and one order of magnitude more potent against hBuChE. Furthermore, nitidine also showed significant, although weak, antiaggregating activity towards amyloid-β self-aggregation.

## 1. Introduction

Since ancient times, plants have been known as a source of active compounds either with poisoning or therapeutic effects. Traditional herbal medicine (THM) lays on the idea that the therapeutic effect of an herbal drug is related to the peculiar mixture of phytocomponents which synergistically exert different activities, also possibly improving the solubility of less soluble components [1,2,3,4,5]. On the other hand, plant extracts also represent a vast source of chemically and structurally diverse scaffolds. Challenges related to the execution of biological screening campaigns with a suitably high throughput as well as to the isolation of single phytocomponents in a satisfactory amount have led big pharmaceutical companies to reduce their research and development expenditures in this sector [6,7]. Nevertheless, the structural and chemical diversity of natural products, unparalleled by any synthetic library, makes natural extracts a virtually limitless source of novel lead compounds [8]. Indeed, natural products or their derivatives have contributed to about a third of FDA-approved drugs over the past 20 years [5], and several natural substances are under preclinical and clinical evaluation for the treatment of different pathologies, including Alzheimer’s disease (AD) [9]. AD is a progressive neurodegenerative pathology and the major cause of dementia in elderly people. Despite an extensive search for new pharmacological treatments, only a few drugs are currently available on the market for AD treatment, among which cholinesterase inhibitors (ChEIs) are the major class [10,11]. ChEIs show their beneficial effects on cognition, by inhibiting the breakdown of the neurotransmitter acetylcholine and enhancing the cholinergic tone in brain areas in which this tone is impaired. Hence, administration of anticholinesterase agents temporarily improves cognition and quality of life in patients suffering from AD. Several compounds isolated from plants, animals and microorganisms have shown beneficial and promising effects for the treatment of AD [9]. Among these, the alkaloid galanthamine, isolated from *Galanthus caucasicus*, *Galanthus woronowii* and other Amaryllidaceae, is currently on the market for AD treatment [12].

Looking for activity beyond the traditional use and in the search of new scaffolds for new generations of anticholinesterase compounds, eventually endowed with other beneficial activities, a single concentration screening campaign was carried out on eight Cameroonian plant extracts, which are used as remedies for a wide array of diseases in traditional folk medicine. Plants were collected by investigators from the Department of Drug Sciences University of Pavia, in the camps of Abing in Cameroon and screened by Ellman’s assay [13]. This study was carried out as part of a joint research campaign aimed at identifying active components behind the traditional use as well as at the identification of phytocomponents with promising activities for alternative applications. Within this study, the aqueous decoction from the stem bark of *Phyllanthus muellarianus* (PM) was identified as a potential source of phytocomponents endowed with anticholinesterase activity. In particular, this extract showed an interesting activity profile on both human acetylcholinesterase (hAChE) and butyrylcholinesterase (hBuChE), with inhibition percentages of 22.6 ± 0.6% (n = 4) and 51.4 ± 0.8% (n = 4), respectively, when screened at 100 mg mL^−1^. 

*Phyllanthus muellerianus* (Kuntze) Exell. is a monoecious, glabrous or climbing shrub widespread in the tropical region of West Africa and used in folk African medicine to treat a variety of pathological conditions [14,15,16]. An extensive chemical investigation has been carried out along the years by few research groups to validate the traditional use of PM extracts in the treatment of wound infections and as wound-healing lotions [15,17,18,19,20,21,22,23]. These investigations have led to the identification of the alkaloid nitidine as the main phytocomponent endowed with antimicrobial activity in the stem bark extracts [18], while geraniin was identified as the major antimicrobial agent in the aqueous extracts from aerial part [20]. Finally, (E)-isoelemicin was identified as the major antibacterial agent in stem bark essential oil [17].

Extracts from PM have no indication for the treatment of dementia or cognitive dysfunctions in folk medicine. The only reported traditional application for neurological disorders refers to the use of extracts from PM leaves in folk Nigerian medicine [24]. Hence, limited information is available for possible bioactive compounds with anti-amnesic activity. To our knowledge, two studies were carried out to purposely investigate potential effects of extracts from plants of the *Phyllanthus* genus on memory. The first was published in 2007 by Joshi et al. [25], who showed that the aqueous extracts of *Phyllanthus amarus* leaves and stems could exert antiamnesic activity in mice. This effect was correlated to the anticholinesterase, antioxidant, and anti-inflammatory activity of some phytocomponents which, however, were not identified. The second study was performed in *Phyllanthus niruri* in 2014 by Koay et al. [26]. In the leaf extract, the authors identified isocorilagin, a tannin with cholinesterase inhibitory activity.

No study has been carried out with the same purpose on PM; however, it is worth noting that studies by Boakye et al. highlighted that the aqueous leaf extract of PM can exert anti-inflammatory [27] and antioxidant [28] activities. These properties have been correlated to the presence of geraniin and other ellagitannins. Hence, notwithstanding that these properties are of interest for the treatment of AD in which neuroinflammation [29,30] and oxidative stress [31,32] play key roles, possible limitations in the blood–brain barrier (BBB) permeability of active phytocomponents need to be taken into consideration.

In this study, we report the evaluation of the anticholinesterase activity of extracts from the stem bark of PM and the bio-guided fractionation of the most active extract to identify phytocomponents endowed with anticholinesterase activity. To this aim, flash chromatography, solid phase extraction (SPE), high-performance liquid chromatography (HPLC), and in vitro bioassays were combined to isolate phytocomponents, which were subsequently identified by mass spectrometry (MS) and nuclear magnetic resonance (NMR) analysis. For the most interesting phytocomponents, the inhibitory activity toward amyloid-beta (Aβ) self-aggregation, a well-known pathological hallmark of AD and, also, a widely studied therapeutic target for AD treatment [33], was also evaluated.

## 2. Results and Discussion

Water extracts of the stem bark of PM (PMWE), prepared in accordance with the traditional use (decoction), were selected as a potential source of phytocomponents endowed with anticholinesterase activity within a screening campaign on decoctions from eight African plants collected in the camps of Abing (Cameroon) (Table S1, Supplementary Material). Hence, in the search for phytocomponents with pharmacological activities beyond the traditional use and aiming at assaying most diverse compounds, methanol extracts (PMME) with and without defatting treatment (defatted PMME—dPMME) were also prepared and their activity against hAChE and hBuChE was first evaluated in a single concentration screening (0.10 mg mL^−1^) assay using the colorimetric method developed by Ellman [13]. Treatment with dichloromethane before methanol extraction was carried out to eliminate the more lipophilic components and make the extract more suitable for biological tests in aqueous buffered solutions. All tested extracts were able to significantly inhibit both hAChE and hBuChE (Table 1). The most active extract was dPMME, which inhibited hAChE activity by 52.6 ± 0.6% and hBuChE activity by 70.1 ± 3.1% (Table 1). Comparison of the chromatographic profiles of PMWE and PMME extracts (Figure S1, Supplementary Material) showed a higher complexity for PMME which, nevertheless, seems also to include most of the phytocomponents present in PMWE. Because of the high sample complexity, chromatographic separation was achieved using a monolith column (Performance RP-18e, 100 × 4.6 mm i.d), which granted high efficiency (narrower peaks) and low back pressure. 

Removal of the most lipophilic compounds did not affect the anti-ChE activity while simplifying the chromatographic profile (Figure S1, Supplementary Material). Phytochemical analyses [15] of these three extracts suggested the presence of a significant percentage of polyphenols. In particular, the polyphenol content was 12.6% in PMWE, 21.8% for PMME and 23.7% for dPMME, respectively.

**Figure 1:**
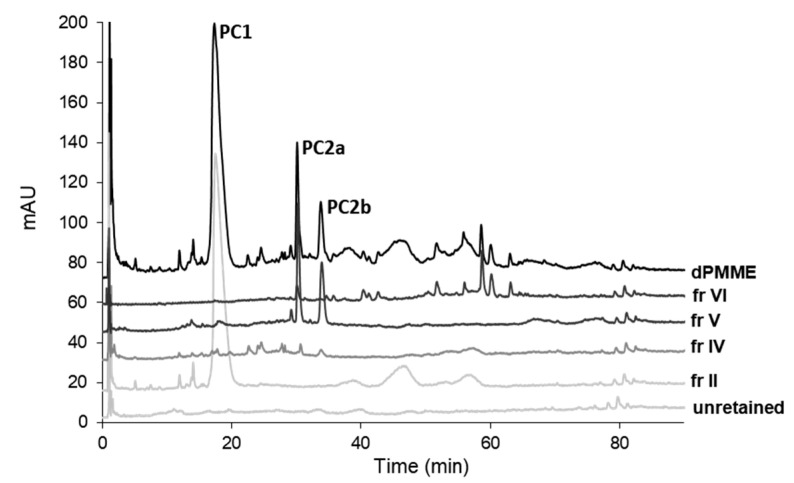
Chromatographic profiles of fractions resulting from dPMME fractionation by SPE on STRATA-X cartridges. Injection volume 20 µL; detection at λ = 220 nm; chromatographic conditions are as detailed in Section 3.6.

In pharmaceutical applications, polyphenols are considered privileged structures capable of interacting with different molecular targets and endowed with antioxidant properties, which can be beneficial for the treatment of various diseases, including AD, in which oxidative stress is enhanced [34]. Based on the preliminary activity screening, a bio-guided fractionation of dPMME on an analytical scale was undertaken in order to facilitate the identification of the components responsible for anti-cholinesterase activity. In order to simplify extract composition prior to further fractionation and facilitate the setting up of the SPE extraction, water solubility of dPMME at 2.0 mg mL^−1^ was assayed. Insoluble fraction was separated by centrifugation, resolubilized in methanol (same initial volume to achieve a concentration identical to the starting solution), analyzed by HPLC (Figure S2, Supplementary Material) and assayed for activity by Ellman’s assay [13]. Comparison of the anti-cholinesterase activities of the water-soluble and water-insoluble fractions of dPMME (Table 2) showed that phytocomponents endowed with anticholinesterase activity are mostly water soluble. Nevertheless, the water-insoluble fraction was endowed with weak but significant anti-BuChE activity (28.1%, n = 3). Chromatographic profiles also showed that most of the phytocomponents in dPMME are water soluble at 2.0 mg mL^−1^ (Figure S2, Supplementary Material). The aqueous solution of dPMME was then subjected to fractionation by SPE on an analytical STRATA-X reversed phase cartridge (Phenomenex). The fractionation process was finely optimized to obtain fractions enriched with individual phytocomponents and to univocally identify the fractions containing major phytocomponents endowed with anticholinesterase activity. In the optimized conditions, six subfractions, named Subfractions I–VI, were isolated. Subfractions I–VI and the loading solution containing unretained compounds were tested by Ellman’s assay [13] at a normalized concentration, i.e., after dilution to the initial loaded volume to assess the contribution of each fraction to the activity of the whole extract (Table 2), and analyzed by HPLC (Figure 1).

**Figure 2:**
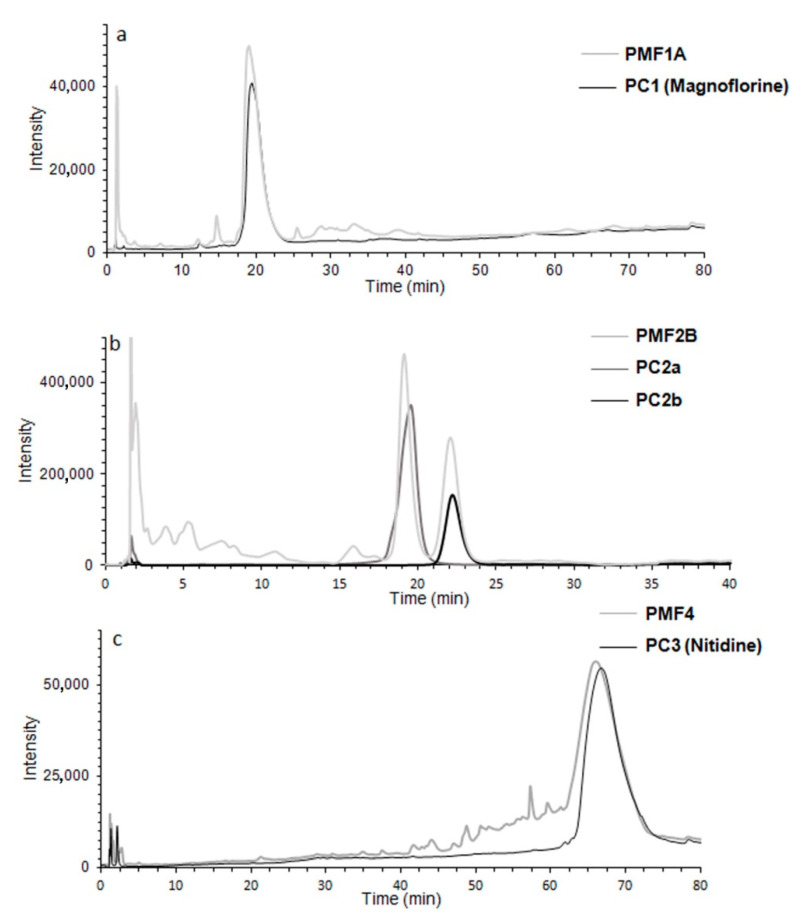
Chromatographic profiles of (**a**) PC1, (**b**) PC2a and PC2b and (**c**) PC3. The chromatographic profile of each isolated phytocomponents is overlaid with that of the PM fraction from which it was isolated, i.e., PMF1, PMF2 and PMF4, respectively.

The most interesting clue that emerges from the analysis of the results (Table 2) is that the phytocomponents with different enzyme selectivity contribute to the overall anticholinesterase activity of dPMME. Indeed, while Subfraction V contains phytocomponents which mainly contribute to the anti-AChE activity of dPMME, Subfraction II contains phytocomponents which exert the highest inhibitory activity on hBuChE. To note, components of Fractions I and V also show a significant anti-BuChE activity. Subfraction II, mostly active on hBuChE, contains a major phytocomponent characterized by an elution time of 16 min (named phytocomponents 1—PC1), which approximately accounts for the 68.7% of the fraction. Subfraction V, mostly active on hAChE, contained two main phytocomponents characterized by a retention times (t_r_) of 30 min (PC2a) and 34 min (PC2b) which account for the 32.9% and 25.4% of the fraction content, respectively. Even if, in the optimized SPE conditions, >95% of PC1 elutes in Subfraction II, Subfraction I also contains a small amount of PC1. It might, therefore, be speculated that PC1, if active, might account for the much weaker anti-BuChE activity exerted by Subfraction I. Hence, in order to collect PC1, PC2a and PC2b in a larger amount, dPMME was fractionated by preparative flash chromatography using a Snap 120 gKP-C18-HS cartridge and a step elution gradient. Out of the six collected fractions (PMF1–6), PMF1, PMF2 and PMF4 significantly inhibited human cholinesterases (Table 3). PMF1 contains the phytocomponent PC1, while PMF2 contains PC2a and PC2b. Hence, these fractions were used as starting material for the isolation of single phytocomponents. Chromatographic profiles of Fractions PMF1, PMF2 and PMF4 are shown in Figure 2a–c. For these fractions, IC_50_ values for cholinesterase inhibition were also determined (Table 3).

A suitable amount of PC1 was isolated by SPE using a Waters Sep-Pak^®^ Vac 12cc C18–2g cartridge while PC2a and PC2b were collected from PMF2 using a re-optimized elution gradient (Section 3.8) able to grant the required peak resolution to get pure compounds. The purity of all collected phytocomponents was confirmed by HPLC analysis (Figure 2a–c) and resulted higher than 95%.

**Figure 3:**
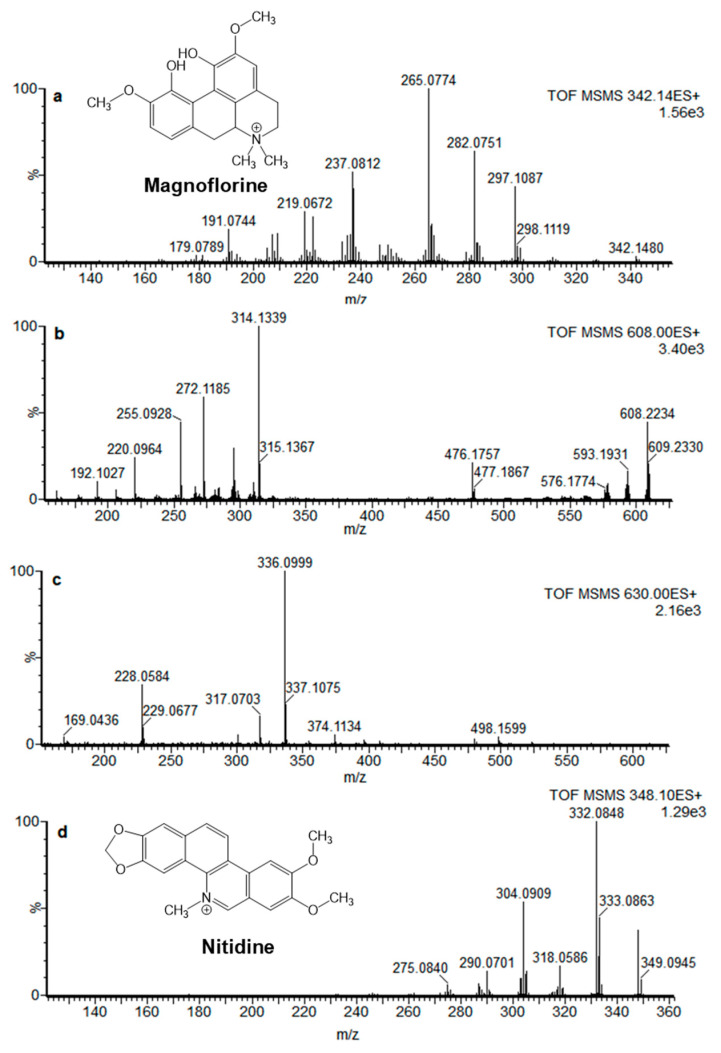
Product ion mass spectra of (**a**) PC1, (**b**) PC2a, (**c**), PC2b, and (**d**) PC3 fraction diluted in methanol and achieved by directed infusion on LC-ESI-Q-TOF. PC1 was identified as magnoflorine, while PC3 was identified as nitidine.

^1^H-NMR (Figure S3, Supplementary Material) and MS analyses were performed on isolated PC1. MS analysis confirmed the high purity of this fraction and showed a molecular peak [M + H]^+^ at *m*/*z* 342.7 (Figure 3a). A literature search combined with mining in MetFrag web-tool (KEGG’s database) [35] led to the identification of magnoflorine. Specifically, magnoflorine’s MS fragment ions were previously reported by Chena et al. [36], while NMR data were available from a publication by Deuk et al. [37]. Identical retention times and MS spectra were obtained for PC1 and magnoflorine commercial standard as further confirmation of the identification. When assayed by Ellman’s method, magnoflorine (structure depicted in Figure 3) showed to act as selective, although weak, hBuChE inhibitor (activity in the two-digit micromolar range) in agreement with the selectivity profile of the fraction PMF1A (Table 4). Other minor phytocomponents of this fraction might also contribute to the slightly higher inhibitory potency of PMF1A. Magnoflorine is an isoquinoline alkaloid that has been found in several plant species [38]. An interesting in vivo study on the anti-aging and anti-AD properties of magnoflorine from Berberis cretica was carried out by Kukula-Koch et al. in 2017 [39]. In this study, administration of a single dose (20 mg kg^−1^ b.w.) of magnoflorine significantly enhanced cognitive functions of male Swiss mice in a passive avoidance test and was able to reverse long-term scopolamine-induced memory impairment [39]. Molecular mechanisms of this activity were not investigated; however, since magnoflorine is predicted to be able to cross the BBB [39], it might be speculated that this pro-cognitive action might be partially correlated to its anticholinesterase activity at a central level. Concerning PMF2, MSMS spectra of the two major phytocomponents were acquired (Figure 3b,c for PC2a and PC2b, respectively); however, data search in web databases (i.e. MetFrag [35] and GNPS Library [40]) did not lead to an univocal identification. Furthermore, the evaluation of the anticholinesterase activity of the two major phytocomponents of this fraction showed that neither PC2a nor PC2b was able to significantly inhibit ChEs. Hence, the anti-ChE activity of PMF2 might arise from the synergic activity of multiple minor phytocomponents. 

Finally, the activity screening of PMF1-PMF6 led to the identification of a further active fraction, namely, PMF4. This fraction contains a major phytocomponent (PC3) which is present in low concentrations in the unfractionated dPMME; hence, it was missed in the preliminary analytical SPE fractionation. However, preparative flash chromatography allowed for the collection of a fraction in which PC3 is the major phytocomponent. This fraction, i.e., PMF4, showed high inhibitory potency towards both ChE enzymes (Table 3) with a slight selectivity for hAChE. The major active PC from F4 was collected using a procedure similar to that used for phytocomponents from PMF2 (Figure 2c) and was identified as nitidine by comparison of its MSMS spectrum (Figure 3d) with those acquired in previous studies on PM extracts by some of the authors [18]. Nitidine was shown to be a good ChEI with comparable inhibitory potencies against both enzymes (Table 4), in agreement with the selectivity trend observed for PMF4.

Nitidine (2,3-dimethoxy-12-methyl-[1,3]benzodioxolo[5,6-c]phenanthridin-12-ium) is a quaternary ammonium alkaloid, which provides multiple biological activities [41,42] and has been previously identified as the main phytocomponent responsible for the traditional use of stem bark decoctions for wound infections by Baka Pigmies [18]. The simultaneous occurrence of magnoflorine and nitidine in a plant extract is not uncommon [43]. These secondary metabolites, belonging to the isoquinoline alkaloids family, share the same biosynthetic pathway, which has phenylalanine and tyrosine as precursors [44]. Nitidine is a benzophenanthridine derivative whose chemical structure (Figure 3) is closely related to that of the alkaloid chelerythrine (1,2-dimethoxy-12-methyl[1,3]benzodioxolo[5,6-c]phenanthridin-12-ium) from Chelidonium majus. The two compounds only differ for the position of the methoxy groups on the benzophenathridin scaffold. In a study by Brunhofer et al., chelerythrine was identified as micromolar inhibitor of human cholinesterases [45], similar to what was observed for nitidine in this study. In the study by Brunhofer et al., chelerythrine also showed an ability to significantly inhibit Aβ self-aggregation [45], which is a known pathological player in Alzheimer’s disease [33]. Hence, based on the structural similarity, the antiaggregating properties of nitidine were also evaluated using a Thioflavin T fluorescence assay [46]. Nitidine acted as weak inhibitor of 42-aminoacid long Aβ peptide (Aβ_1–42_) self-aggregation with an inhibition percentage of 15.9 ± 1.0 when assayed at a 1:1 molar ratio with Aβ.

## 3. Materials and Methods

### 3.1. Plant Material

The stem bark of *Phyllanthus muellerianus* was collected in Cameroon in July 2009 in the camps of Abing. The plant was identified at the National Herbarium of Yaoundé by the Cameroonian botanist Mr. Nana. A voucher specimen (no. BWPV03) has also been deposited at the Department of Drug Sciences of the University of Pavia. Bark was dried in the dark, in a ventilate room at 25–30 °C, then ground and the powder stored at −20 °C.

### 3.2. Extraction Procedure

Extraction procedure was carried out as previously reported in [15]. The dried powder (100 g) was refluxed in distilled water (700 mL) for 3 h, and the crude extract obtained was frozen and lyophilized (PMWE). Yield of extraction = 2.4% on a dry mass basis. Further extractions were performed in MeOH (PMME). A total of 25 g of dried powder was suspended in 100 mL of methanol. The mixture was refluxed for 60 min, filtered, re-suspended in 100 mL of fresh methanol and refluxed for further 60 min (3 times). The fractions were collected, and solvent was removed under vacuum. Yield of extraction for PMME was 3.8% on a dry mass basis. The defatted methanol extract (dPMME) was prepared following the same procedure described above but using dichloromethane (3 × 100 mL) before methanol (3 × 100 mL). Yield of extraction of the dPMME fraction was 0.8% on a dry mass basis. Dried extracts were stored at −20 °C until biological tests.

### 3.3. Determination of Polyphenol Content

The total polyphenol content was determined by the slightly modified spectrophotometric method by the Folin–Ciocalteu method [47] as previously reported in [48]. Briefly, 10–50 μL of each extract was transferred into a test tube containing 6 mL of water and 0.5 mL of tenfold diluted Folin–Ciocalteu reagent. The mixture was vortexed and allowed to stand at room temperature for 3 min before the addition of 1.5 mL of 20% *w*/*v* sodium carbonate aqueous solution. Then, the mixture was kept at room temperature for a further 2 h before the absorbance at 760 nm was recorded. In order to quantitate total polyphenol content, a calibration curve was prepared using gallic (1–3 mg/L) as a reference standard and following the same protocol described for samples. All measurements were carried in 1 cm path length disposable plastic cuvettes (Ettore Pasquali S.r.l., Milan, Italy) at 760 nm. Experiments were carried out in triplicate. The total polyphenol content was expressed as gallic acid percentage, refereed to the dry weight powder.

### 3.4. Fractionation of dPMME by Solid Phase Extraction

An exactly weighted amount of dPMME was solubilized in distilled water to get a 2 mg mL^−1^ solution which was vortexed, sonicated, and centrifuged to remove the water-insoluble fraction. To evaluate fraction composition by HPLC (see Section 3.6) and anticholinesterase activity (Section 3.12), the insoluble residue was re-solubilized in an appropriate volume of methanol to re-achieve the initial concentration. Water solution of dPPME was subjected to SPE using STRATA-X cartridges 30 mg/1 mL (Phenomenex, Castel Maggiore (BO), Italy) and a 10-position VacMaster system (International Sorbent Technology LTD, Hengoed, U.K.). Cartridges were activated by methanol (2 mL) and conditioned by bidistilled water (2 mL). Loaded sample volume was 3.5 mL (unretained fraction). Fractionation was achieved by sequentially adding 0.5 mL of H_2_O/MeOH 90/10 (*v*/*v*)—Subfraction I, 1 mL H_2_O/MeOH 75/25 (*v*/*v*) —Subfraction II, 0.5 mL H_2_O/MeOH 75/25 (*v*/*v*)—Subfraction III, 1 mL of H_2_O/MeOH 60/40 (*v*/*v*)—Subfraction IV, 1 mL of H_2_O/MeOH 45/55, (*v*/*v*)—Subfraction V, 1 mL MeOH—Subfraction VI. SPE fractions I–IV were stored as solutions at −80°C until use. All the fractions were tested for their anticholinesterase activity as described in Section 3.12 and analyzed by HPLC as in Section 3.6. 

### 3.5. Fractionation of PMME by Flash Chromatography

The dPPME (2 g) was fractionated by flash chromatography using a Biotage Isolera Prime system and a Snap 120 gKP-C18-HS cartridge. A stepwise gradient H_2_O-AcCN with 0.08% of TFA was used. Gradient elution: 95/5 → 90/10 in 2 CV (column volume = 150 mL, each achieved in 3.75 min), 90/10 → 88/12 in 3 CV, 88/12 in 5 CV, 88/12 → 85/15 in 15 CV, 85/15 → 72/28 in 13 CV, 72/28 in 10 CV, 72/28 → 50/50 in 5 CV, 50/50 → 30/70 in 5 CV, 30/70 → 100 in 7 CV, at a flow rate of 40 mL min^−1^. The elution was monitored at 225 and 366 nm. Six fractions were collected, named PMF1-PMF6. Yields for PMF1-PMF6 isolation were: 18.0%, 7.2%, 7.3%, 8.7%, 1.4%, 13.3% from PMF1 to PMF6, respectively. Isolated fractions were subjected to HPLC analysis and assayed as reported in Section 3.12. Fractions were assayed for anticholinesterase activity, and PMF1, PMF2 and PMF4 were used to isolate major phytocomponents.

### 3.6. Chromatographic Analysis

HPLC-UV analyses were carried out by a Hewlett Packard HP-Ti-1050-Series chromatographic system using a C18 monolithic column (Chromolith Performance RP-18e, 100 × 4.6 mm i.d., Merck KGaA, Darmstadt, Germany). Chromatograms were recorded at 220 nm. Bidistilled water and acetonitrile (AcCN) were used as mobile phase A and B, respectively. A gradient was set as follows: 0–6 min 3% B; 6–15 min from 3 to 7.8% B; 15–21 min from 7.8 to 9% B; 21–27 min from 9 to 13.5% B; 27–37 min from 13.5 to 15.75% B; 37–44 from 15.75 to 18% B; 44–60 min from 18 to 29.1% B; 60–80 min from 29.1 to 47.6% B; 80–82 min from 47.6 to 55% B; flow rate from 0 to 82 min was 1.4 mL/min; 82–83 min 55% B, flow rate increased from 1.4 to 2.0 mL/min; 83–90 min 55% B, flow rate = 2.0 mL/min. The column was re-equilibrated with the starting conditions of analysis for 10 min before the next injection.

### 3.7. Isolation of PC1 from F1 

A total of 70 mg of PMF1 was solubilized in 1 mL of distilled water. The solution was sonicated for 5 min before being subjected to SPE on a Waters Sep-Pak^®^ Vac 12cc C18—2 g cartridge. The cartridge was activated by 40 mL of acetonitrile (AcCN) containing 0.08% of trifluoroacetic acid (TFA), and conditioned by 40 mL of H_2_O/AcCN 95/5 (*v*/*v*) with 0.08% TFA. Fractionation was achieved by sequentially adding 40 mL of H_2_O/AcCN 95/5 (*v/v*) with 0.08% TFA 95/5 (*v/v*) (Subfraction I), 40 mL H_2_O/AcCN 90/10 (*v/v*) with 0.08% TFA (Subfraction II), 40 mL of H_2_O/AcCN 85/15 (*v/v*) with 0.08% TFA (Subfraction III), 40 mL of H_2_O/AcCN 80/20 (*v/v*) with 0.08 TFA (Subfraction IV), 20 mL of H_2_O/AcCN 50/50 (*v/v*) with 0.08% TFA (Subfraction V), and 20 mL H_2_O/AcCN 0/100 (*v/v*) with 0.08% TFA (Subfraction VI). Fraction III contains PC1 as determined by chromatographic analysis of all fractions. PC1 fraction was stored at −80 °C until use. Isolation yield = 16.7% on a dry mass basis.

### 3.8. Isolation of PC2a and PC2b from Fraction F2

Based on the biological activity, F2 was chromatographed on a C18 monolithic column (Chromolith Performance RP-18e, 100 × 4.6mm i.d., Merck KGaA, Darmstadt, Germany) in order to collect PC2a and PC2b. Analyses were carried out by a Hewlett Packard HP-Ti-1050-Series chromatographic system using a C18 monolithic column (Chromolith Performance RP-18e, 100 × 4.6 mm i.d., Merck KGaA, Darmstadt, Germany). Bidistilled water and AcCN were used as mobile phase A and B, respectively. The gradient conditions were set as follows: 0–35 min from 10% B to 20% B, 35–45 min from 20% B to 50% B. Flow rate was 1.0 mL min^−1^. Column was re-equilibrated with starting conditions for 15 min before the following injections. Subsequent injections of a 4.0 mg/mL solution of fraction F2 (100 μL) were performed. Eluates corresponding to PC2a and PC2b were collected in volumetric flasks and subjected to SPE extraction on STRATA-X SPE cartridge in order to carry out a solvent exchange and concentrate single phytocomponents. Prior SPE extraction collected eluates were diluted 1.5 times with bidistilled water to lower the concentration of AcCN to about 10%, thus preventing elution of the isolated phytocomponents during the loading step. SPE steps were set as follows: activation 2 mL MeOH; conditioning 3 mL bidistilled water; loading 7 mL phytocomponent solution; washing 1 mL bidistilled water; elution 1 mL MeOH. Purity was assessed by HPLC analysis in the same conditions used for phytocomponent collection. Isolated phytocomponents were subjected to MS analysis as reported in Section 3.10 and to the evaluation of the anticholinesterase activity.

### 3.9. Isolation of PC3 from Fraction F4

F4 was chromatographed on a C18 monolithic column (Chromolith Performance RP-18e, 100 × 4.6 mm i.d., Merck KGaA, Darmstadt, Germany) in order to collect PC3. Chromatographic conditions were as in Section 3.5. Isolation yield = 21.5% on a dry mass basis.

### 3.10. MS Analysis of Isolated Phytocomponents

PC1 isolated from PMF1, PC2a and PC2b from PMF2 and PC3 from PMF4 fraction were diluted in methanol and directly infused in an ESI-Q-ToF mass spectrometer equipped with a Z-spray ion source (Micromass, Manchester, UK) at the flow rate of 10 µL min^−1^ using a syringe pump. The ion source temperature was set at 120 °C with a cone gas flow of 80 L h^−1^, a desolvation gas temperature of 300 °C and a desolvation gas flow of 600 L h^−1^. The capillary voltage was set at 3.0 kV in positive ion mode. A scan time of 1 s with an inter-scan delay of 0.1 s was used. Fragmentation voltage was set at 30 eV for the MSMS analysis of PC1 parent ion (*m/z* 342.14), at 40 eV for PC2a and PC2b parent ions (*m/z* 608.22 and 630.19, respectively) and at 35 eV for PC3 parent ion (*m/z* 348.10). The identity of PC1 and PC3 was confirmed by the direct infusion and ESI-MSMS analysis of magnoflorine and nitidine standards under the same experimental conditions.

### 3.11. ^1^H-NMR Analysis

^1^H-NMR spectra were recorded on a Varian Inova 500 MHz NMR Spectrometer (International Equipment Trading Ltd, Mundelein, IL, USA) using DMSO as solvent and TMS as an internal standard. 

### 3.12. Inhibition of Human Cholinesterase Activity

Human recombinant AChE and BuChE from human serum and all reagents, substrates and salts used for the assay were purchased from Sigma-Aldrich (Milan, Italy). Nitidine and magnoflorine as reference standards were purchased from Sigma Aldrich. Ellman’s assay [13] was followed with minor modifications. hAChE stock solution was prepared in potassium phosphate buffer 0.1 M containing Triton 0.1% (*v/v*). hBuChE stock solution was prepared in potassium phosphate buffer 0.1 M. Acetyl, and butyrylthiocholine iodide 34.6 mM stock solutions were prepared in deionized water, aliquoted and stored at −20 °C until use. Assay solution contained 0.1 M potassium phosphate buffer (pH 8.0), 340 µM DTNB, and PM extracts/fractions and 0.02 unit mL^−1^ of enzyme. Blank solutions containing all components except the ChE enzyme were prepared in parallel to account for the non-enzymatic hydrolysis of the substrate. Prior activity determination, samples and blanks were incubated for 20 min at 37 °C in 1.2 mL polystyrene cuvettes (Hellma, Milan, Italy). Reaction was started by adding the substrate at the final concentration of 554 µM. The formation of the chromophore was monitored at 412 nm for 200 s by a Jasco V-530 spectrophotometer (Jasco Europe, Cremella, Italy) equipped with a thermostated cuvette holder (T = 37 °C). Comparing the enzymatic activity in the presence (AE_i_) and in the absence (AE_0_) of PM extracts or subfractions, the percentage of residual enzyme activity (% AE_r_) and the percentage of inhibition were derived. All extracts and fractions were initially assayed at a single concentration (100 µg mL^−1^). For IC_50_ determination, five increasing concentrations of the tested solution were used, able to give an inhibition of the enzymatic activity in the range of 20–80%. The results were plotted by placing the percentage of inhibition in the function of the decimal log of the final inhibitor concentration. Linear regression and IC_50_ values were calculated using Microcal Origin software (Microcal Software, Inc., version 3.5).

### 3.13. Inhibition of Aβ_1–42_ Self-Aggregation

As reported in the previously published protocol by Bartolini et al. [46], 1,1,1,3,3,3,hexafluoro 2-propanol pre-treated Aβ_1–42_ samples (Bachem AG, Bubendorf, Switzerland) were solubilized with a AcCN/0.3 mM Na_2_CO_3_/250 mM NaOH (48.4/48.4/3.2) mixture to obtain a 500 μM stock solution. Experiments were performed by diluting (final Aβ concentration 50 μM) and incubating the peptide in 10 mM phosphate buffer (pH = 8.0) containing 10 mM NaCl, at 30 °C for 24 h with and without nitidine (50 μM, Aβ/nitidine = 1/1). A blank sample containing nitidine was also prepared. To quantify amyloid fibril formation, the thioflavin T fluorescence method was used [49]. After incubation, samples were diluted to a final volume of 2.0 mL with 50 mM glycine-NaOH buffer (pH 8.5) containing 1.5 μM thioflavin T. A 300-second time scan of fluorescence intensity was carried out (λexc = 446 nm; λem = 490 nm, FP-6200 fluorometer, Jasco Europe, Cremella, Italy), and values at plateau were averaged after subtracting the background fluorescence of 1.5 μM thioflavin T solution. The fluorescence intensities obtained in the absence and in the presence of nitidine were compared and the percent inhibition due to the presence of nitidine was calculated by the following formula: 100 − (100 × IFi/IFo), where IFi and IFo are the fluorescence intensities obtained for Aβ_1–42_ in the presence and in the absence of nitidine, respectively.

## 4. Conclusions

The activity of dPMME on human ChEs is related to the presence of at least two active phytocomponents, namely, magnoflorine and nitidine, which are endowed with different activity and selectivity profiles for the two isoforms of cholinesterase enzymes: magnoflorine is a weak ChE inhibitor, selective for hBuChE (selectivity index = 8.5), while nitidine shows an interesting anticholinesterase activity in the low micromolar range. When compared with the commercially available AD drug galanthamine, nitidine results equally potent on hAChE and one order of magnitude more potent against hBuChE. However, the ammonium group in nitidine may limit its load in the brain. Nevertheless, nitidine scaffold could be used as a starting template to design new naturally inspired ChE inhibitors endowed with multiple activities. Indeed, in the attempt of finding effective treatments for AD, several groups are pursuing the development of multifunctional compounds, i.e., compounds able to interact with more than a single target [50,51]. In the light of this approach and, for example, similar to what was previously pursued with propidium, a phenylphenanthridin derivative and a known binder of AChE at the peripheral binding site [52], nitidine, or even magnoflorine, could be used as a starting template to generate new hybrid molecules with an enlarged activity profile and reduced toxicity. Furthermore, as clearly demonstrated by results from PMF2 and in agreement with the basic concepts of THM, eventually simplified fractions of PM extracts might find application as a whole, their activity likely being the result of a synergic contribution of several components. This aspect might be worthy of further and future investigations.

## Figures and Tables

**Table 1:** Anticholinesterase activity of PM extracts at 0.10 mg/mL.

	hAChE Inhibition % ± SD	hBuChE Inhibition % ± SD
PMWE	22.6 ± 0.6	51.4 ± 0.8
PMME	46.8 ± 1.8	64.1 ± 0.7
dPMME	52.6 ± 0.6	70.1 ± 3.1

Each value is the mean of at least two independent determinations, each carried out in triplicate. PMWE = *Phyllanthus muellarianus* water extract; PMME = *Phyllanthus muellarianus* methanol extract; dPMME = defatted PMME.

**Table 2:** Inhibitory activity of Subfractions I–VI obtained by SPE separation from the water-soluble fraction of dPMME towards human cholinesterase enzymes. Activity of the whole dPMME extract and water-insoluble fractions are also listed.

dPMME	Composition of the Eluting Solution	Inhibition hAChE, % ± SD	Inhibition hBuChE, % ± SD
**Whole extract**		52.6 ± 0.6	70.1 ± 3.1
**Water-soluble fraction**		60.4 ± 1.0	74.9 ± 0.9
*Loading solution*	H_2_O	n.a.	18.4 ± 0.9
*Subfraction I*	H_2_O/MeOH 90/10	n.a.	28.3 ± 2.4
*Subfraction II*	H_2_O/MeOH 75/25	**10.4 ± 1.2**	**46.3 ± 5.5**
*Subfraction III*	H_2_O/MeOH 75/25	6.5 ± 1.1	20.2 ± 5.6
*Subfraction IV*	H_2_O/MeOH 60/40	16.2 ± 5.0	13.5 ± 1.3
*Subfraction V*	H_2_O/MeOH 45/55	**42.0 ± 1.7**	**28.2 ± 1.3**
*Subfraction VI*	MeOH	12.0 ± 3.5	13.4 ± 2.5
**Water-insoluble fraction**		18.1 ± 3.0	28.1 ± 3.0

Each value is the mean of at least two independent determinations, each carried out in duplicate. Prior to assay, each subfraction was diluted to the initial loaded volume in order to get the relative contribution of each fraction to the activity of water-soluble components of dPMME (0.10 mg mL^−1^). n.a. stands for not active (% inhibition < 5%).

**Table 3:** Anticholinesterase activity of active fractions achieved by flash chromatography.

Fraction Code	Inhibition hAChE. % ± SD	IC_50_ hAChE, µg/mL	Inhibition hBuChE. % ± SD
PMF1	31.0 ± 0.7	167 ± 8	76.1 ± 1.7
PMF2	52.9 ± 0.3	84.4 ± 5.9	64.6 ± 2.1
PMF4	>90	2.95 ± 0.14	>90

**Table 4:** Anticholinesterase activity of isolated phytocomponents and reference compound galanthamine, a drug in the market for the treatment of Alzheimer Disease.

Compound	Inhibition hAChE at 100 µg/mL % ± SD	IC_50_ hAChE µM ± SD	Inhibition hBuChE at 100 µg/mL % ± SD	IC_50_ hBuChE µM ± SD
Magnoflorine (PC1)	19.0 ± 2.9	1120 ± 83	69.9 ± 2.4	131 ± 9
PC2a	n.a.	n.d.	n.a.	n.d.
PC2b	n.a.	n.d.	n.a.	n.d.
Nitidine (PC3)	>90	5.31 ± 0.50	>90	6.68 ± 0.13
Galanthamine	>90	2.01 ± 0.15	>90	20.7 ± 1.5

n.a. stands for not active (% inhibition < 5%); n.d. stands for not determined.

## Data Availability

Data are contained within the article and Supplementary Material.

## Supplementary Materials

The following are available online, Screening of African plants towards human acetyl—and butyrylcholinesterase: experimental part, Table S1: Anticholinesterase activities expressed as percentage of inhibition of decoctions from African plants collected in Cameroon, Figure S1: Chromatographic profiles of fractions PMWE, PMME and dPMME, Figure S2: Chromatographic profiles of dPMME and its water-soluble and insoluble fractions, Figure S3: ^1^H-NMR spectrum of PC1 extract, identified as magnoflorine.

## Sample Availability

Samples of the dry extracts PMWE, PMME, dPMME are available from the authors.
