# Supplementary material for: Bio-Guided Fractionation of Stem Bark Extracts from Phyllanthus muellarianus: Identification of Phytocomponents with Anti-Cholinesterase Activity
Source: Molecules. 2021 Jul 20;26(14):4376. doi: 10.3390/molecules26144376 (PMC8307647; doi:10.3390/molecules26144376)
Supplement: Supplementary file 1 [file molecules-26-04376-s001.zip › molecules-1266473-supplementary.pdf]

SUPPLEMENTARY MATERIAL

BIO-GUIDED FRACTIONATION OF STEM BARK EXTRACTS FROM *PHYLLANTHUS MUELLERIANUS*: IDENTIFICATION OF PHYTOCOMPONENTS WITH ANTI-CHOLINESTERASE PROPERTIES

Marina Naldi[1,2], Caterina Temporini[3], Gabriella Massolini[3], Vincenza Andrisano[4], Gloria Brusotti[3], Manuela Bartolini[1]

[1]Department of Pharmacy and Biotechnology, Alma Mater Studiorum University of Bologna, Via Belmeloro 6, 40126, Bologna, Italy;

[2]Center for Applied Biomedical Research (CRBA), S. Orsola–Malpighi Hospital, Via Massarenti 9, 40138 Bologna, Italy

[3]Department of Drug Sciences, University of Pavia, Viale Taramelli 12, 27100 Pavia, Italy;

[4]Department for Life Quality Studies, Alma Mater Studiorum University of Bologna, Corso D'Augusto 237, 47921, Rimini, Italy.

Table of contents

Screening of African plants towards human acetyl- and butyrylcholinesterase: experimental part	*S1*
***Table S1**. Anticholinesterase activities expressed as percentage of inhibition of decoctions from African plants collected in Cameroon*	*S2*
***Figure S1.** Chromatographic profiles of fractions PMWE, PMME and dPMME*	*S2*
***Figure S2.** Chromatographic profiles of dPMME and its water soluble and insoluble fractions.*	*S2*
***Figure S3.** ^{1}H-NMR spectrum of PC1 extract, identified as magnoflorine*	*S3*

Screening of African plants towards human acetyl- and butyrylcholinesterase: experimental part

Plant material

Plants were collected in Cameroon in July 2009 in the camps of Abing; after the identification at the National Herbarium of Yaoundé by the Cameroonian botanist Mr. Nana, voucher specimens (no. *Bridelia grandis* BWPV01, *Piptadeniastrum africanum* BWPV02, *Phyllanthus muellerianus* BWPV03, *Panda oleosa* BWPV04, *Parinari excelsa* BWPV05, *Strombosiopsis tetranda* BWPV06, *Trichilia lanata* BWPV07, *Dyospiros bipindensis* BWPV08) were deposited at the Department of Drug Sciences of the University of Pavia.

Extraction procedure

The stem bark dried powder (100g) was refluxed in distilled water (700 mL) for 3 h and the crude extract obtained was frozen and lyophilized.

Results

Table S1. Anticholinesterase activities expressed as percentage of inhibition of decoctions from African plants collected in Cameroon. Anticholinesterase activities were determined by Ellman's method (1) as described in the experimental part (main text).

	**Tested concentration on hAChE (mg/mL)**	**hAChE % inhibition ±SD**	**Tested concentration on hBuChE (mg/mL)**	**hBuChE % inhibition ±SD**
Trichilia lanata	0.10	n.a.	0.10	n.a.
Bridelia grandis	0.025	9.8 ± 2.1%	0.05	n.a.
Panda oleosa	0.05	n.a.	0.10	n.a.
Parinari excelsa	0.10	n.a.	0.025	n.a.
Dyospiros bipindensis	0.10	n.a.	0.10	5.6 ± 1.8
Phyllantus muellarianus	**0.10**	**22.6 ± 0.6**	**0.10**	**51.4 ± 0.8**
Strombosiopsis tetranda	0.05	n.a.	0.025	n.a.
Piptadeniastrum africanum	0.05	n.a.	0.10	n.a.

hAChE: human recombinant acetylcholinesterase; hBuChE: butyrylcholinesterase from human serum; each value is the mean of at least two independent determinations, each carried out in triplicate. n.a. stands for not active (% inhibition <5%).

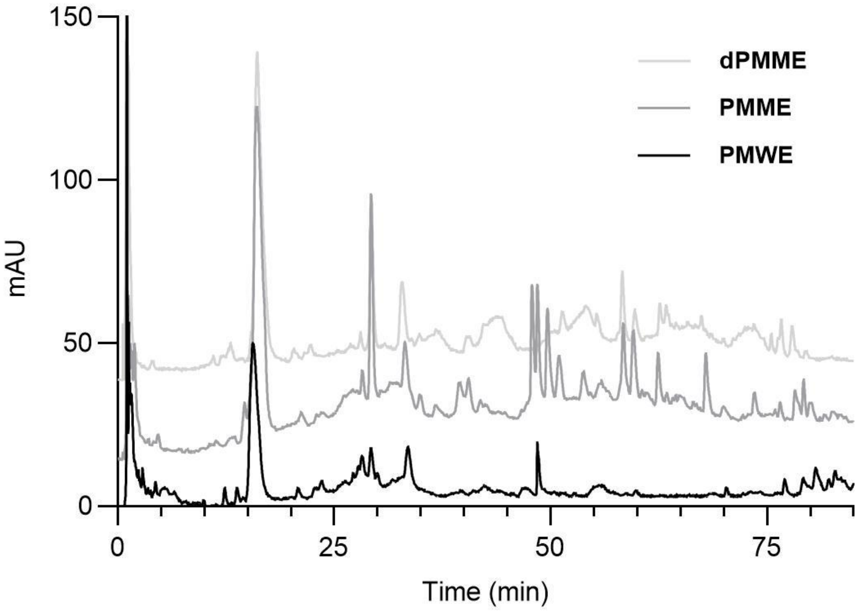

Figure S1. Chromatographic profiles of fractions PMWE, PMME and dPMME. Injection volume 20 µL; detection at λ = 220 nm; chromatographic conditions as detailed in section 3.5.

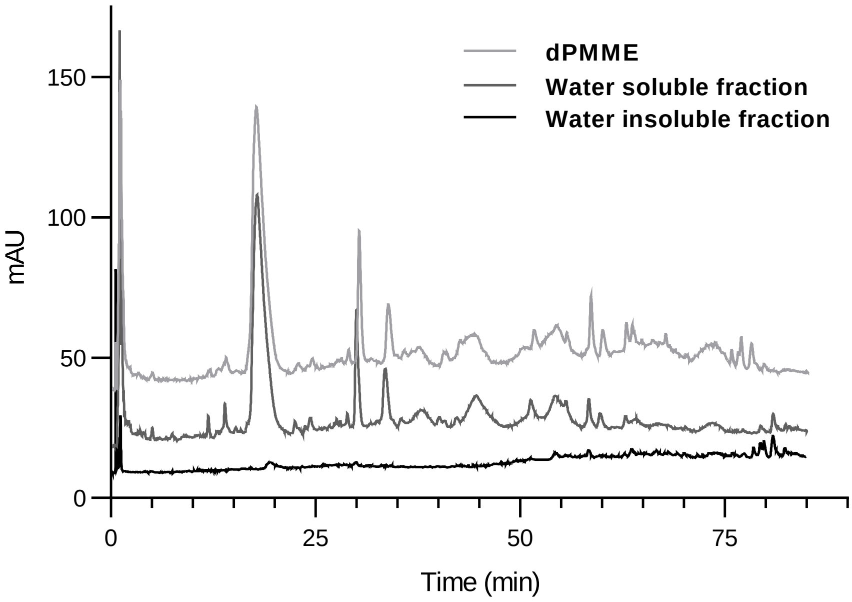

Figure S2. Chromatographic profiles of dPMME and its water soluble and insoluble fractions. Injection volume 20 µL; detection at λ = 220 nm; chromatographic conditions as detailed in section 3.5.

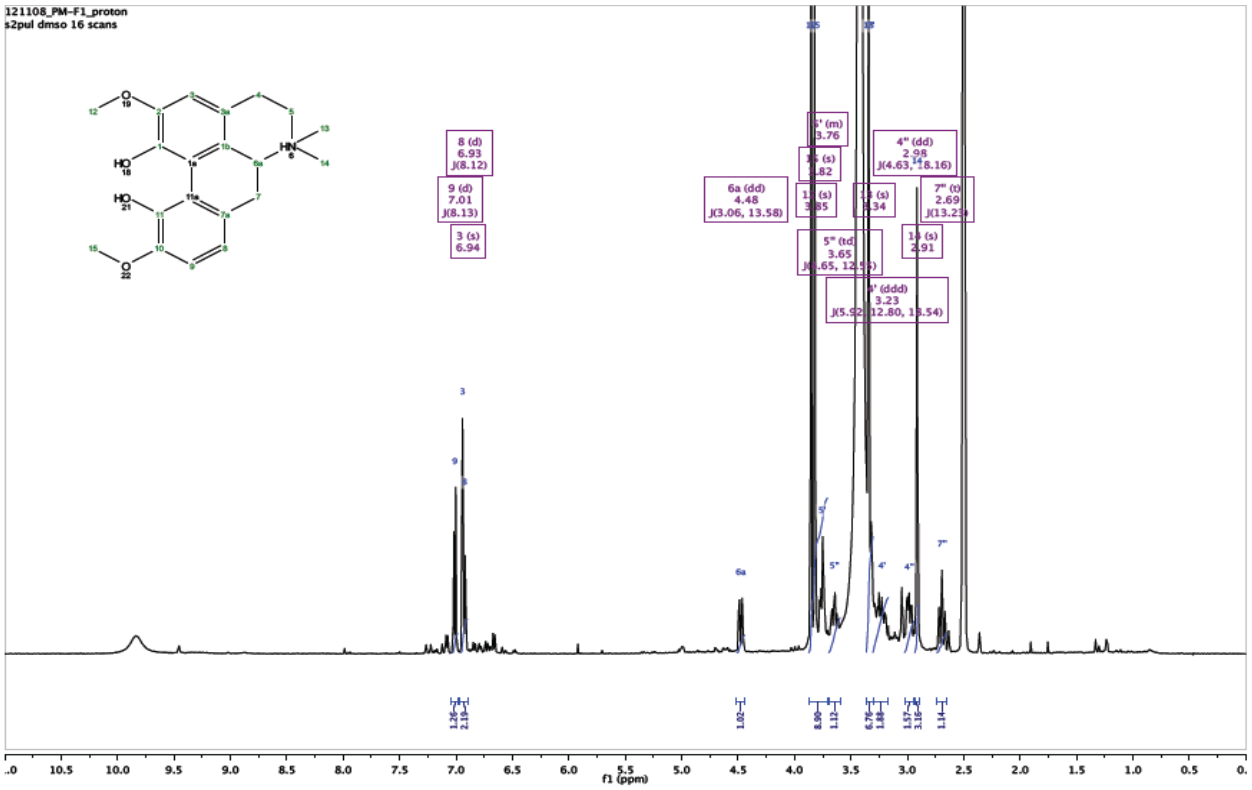

Figure S3. ^{1}H-NMR spectrum of PC1 extract, identified as magnoflorine. Structure of magnoflorine is also depicted.

References

1. Ellman GL, Courtney KD, Andres V, Jr., Feather-Stone RM. A new and rapid colorimetric determination of acetylcholinesterase activity. Biochem Pharmacol. 1961;7:88-95.
